# Short-term associations between ambient air pollution and emergency department visits for Parkinson disease, multiple sclerosis, migraine, and seizure in California, 2005–2018

**DOI:** 10.1097/EE9.0000000000000444

**Published:** 2025-12-23

**Authors:** Shumeng Chen, Danlu Zhang, Rebecca Zhang, Morgan Lane, Noah C. Scovronick, Stefanie T. Ebelt, Howard H. Chang

**Affiliations:** a Department of Biostatistics and Bioinformatics, Emory University, Atlanta, Georgia; b Gangarosa Department of Environmental Health, Emory University, Atlanta, Georgia

**Keywords:** Air pollution, Emergency department visits, Parkinson disease, Multiple sclerosis, Migraine, Seizure, Health effect

## Abstract

**Background:**

Previous studies have linked ambient air pollution to worsening neurological conditions, but research in the United States is limited. This study aims to investigate short-term associations between emergency department (ED) visits for four disorders affecting the nervous system (Parkinson disease, multiple sclerosis, migraine, and seizure) and three major air pollutants: fine particulate matter (PM_2.5_), carbon monoxide (CO), and nitrogen dioxide (NO_2_).

**Methods:**

Daily estimated exposure concentrations at 12 km were aggregated to the ZIP-code level and linked to 6 million ED visits in California (2005–2018). Using a time-stratified case-crossover design with conditional quasi-Poisson regression, we evaluated lag-specific associations and stratified analysis by patient characteristics. Risk ratios (RRs) are reported per interquartile range increase in exposure.

**Results:**

For Parkinson disease, CO showed the strongest short-term association at lag 0 (RR = 1.007, 95% confidence interval [CI]: 1.003, 1.011). No associations were found for multiple sclerosis. For migraines, same-day PM_2.5_ (RR = 1.003, 95% CI: 1.001, 1.005), CO (RR = 1.006, 95% CI: 1.003, 1.008), and NO_2_ (RR = 1.009, 95% CI: 1.005, 1.015) were positively associated with ED visits. For seizures, NO_2_ at lag 0 showed the strongest effect (RR = 1.006, 95% CI: 1.003, 1.010). Compared with same-day exposure, cumulative effects over multiple days decreased for CO and NO_2_ but remained similar for PM_2.5_.

**Conclusion:**

Same-day high ambient air pollution levels were associated with increased ED visits for migraine, Parkinson disease, and seizures. These findings support the growing evidence that short-term air pollution exposure may adversely impact patients with neurological conditions.

**What this study adds**

This study analyzed over 6 million emergency department (ED) visits for four nervous system conditions, including Parkinson disease, multiple sclerosis, seizures, and migraines, and evaluated their short-term associations with exposure to PM_2.5_, NO_2_, and CO. This study represents the largest US investigation linking air pollution to neurological morbidity and uniquely considers both ED visits and hospitalizations. We found that same-day high ambient air pollution levels were associated with increased ED visits for migraine, Parkinson disease, and seizures, but not for multiple sclerosis. This adds to growing epidemiological evidence that short-term exposure to ambient air pollution may affect patients with neurological conditions.

## Introduction

Ambient air pollution is a significant threat to public health, affecting millions of individuals worldwide. Ambient air pollution has been linked to various adverse health outcomes such as cardiovascular diseases (CVDs), respiratory issues, and immune system dysregulation.^1–5^ Recent studies have also reported associations between air pollution and the development, progression, and exacerbation of neurological conditions.^6,7^ Particulate matter (PM), nitrogen dioxide (NO_2_), and carbon monoxide (CO) have been the most widely studied air pollutants. Fine PM with diameters ≤ 2.5 µm (PM_2.5_) can reach the bloodstream through the respiratory system, causing systemic inflammation that may affect the nervous system.^8,9^ PM_2.5_ can also directly enter the brain through olfactory nerves.^10^ NO_2_, primarily produced by vehicle emissions and industrial activities, contributes to oxidative stress and cellular damage.^11^ CO disrupts oxygen transport in the blood, leading to hypoxia and potential neurological effects.^12^ Given the high prevalence of neurological conditions globally^13^ and their considerable impacts on patient quality of life and healthcare burden,^14,15^ understanding risks associated with ambient air pollution is critical for public health.

Parkinson disease (PD), seizures, and other nervous system disorders are particularly susceptible to environmental influences.^16–18^ Several recent studies have investigated both long-term and short-term effects of air pollution exposure. For short-term exposure, in France (2009–2017), PM_10_, PM_2.5_, and NO_2_ were linked to a rise in hospital admissions for PD, particularly during cold and warm seasons.^19^ In a study of European cities, short-term exposure to PM_2.5_ was associated with increased emergency admissions for both Alzheimer disease (AD) and PD, with older individuals being more affected and AD showing stronger effects at shorter lags.^20^ Additionally, in Taiwan (2009–2013), pollutants such as CH_4_, NO, and PM_2.5_ were associated with hospital visits for seizures, with the strongest associations observed during winter.^21^ In Seoul (2008–2014), researchers found that higher air pollution levels were associated with a higher risk of migraines in various lag structures^.22^ And a study in Strasbourg (2000–2009) found that short-term exposures to NO_2_, PM_10_, and O_3_ were associated with an increased risk of multiple sclerosis (MS) relapses.^23^

For long-term exposures, a large nationwide cohort study in the United States reported that PM_2.5_ was associated with increased risks of PD- and AD-related dementias.^24^ Meanwhile, in a large Medicare cohort across northeastern US cities (1999–2010), the study found that long-term PM_2.5_ exposure was significantly associated with increased risks of first hospital admission for dementia, AD, and PD.^25^ A large UK Biobank cohort study found that long-term exposures to multiple air pollutants (PM_2.5_, PM_10_, and NO_x_) were associated with an increased risk of new-onset migraine.^26^ However, a large Canadian population-based cohort study did not find significant associations between long-term exposure to PM_2.5_, NO_2_, or O_3_ and the incidence of MS.^27^

Despite growing evidence from short-term studies of air pollution and neurological conditions such as PD, most prior work has focused on single conditions or non-US populations, and evidence based on emergency department (ED) visits in the United States remains scarce. ED care represents a crucial indicator for disease burden and is an understudied outcome compared with hospital admission and mortality.^28^ The ED is the only component in the US healthcare system that is accessible at any time of day and plays important roles in disaster response and syndromic surveillance.^29–31^ Also, because EDs provide treatment regardless of the ability to pay, they often serve as the safety net for populations with care-seeking barriers.^32–34^ Finally, the role of ambient CO in contributing to acute neurological outcomes remains underexplored despite known pathophysiological mechanisms.

This study aims to investigate associations between short-term exposure to PM_2.5_, CO, and NO_2_ and ED visits for four neurological conditions, including PD, MS, migraine, and seizure, to address these research gaps. These conditions were selected due to their significant public health importance and the limited but emerging evidence linking them to environmental exposures. Together, they represent a significant healthcare burden in the United States—migraine alone affects 1 of every 6 American,^35^ while seizures affect approximately 3.4 million.^36^ MS^37^ and PD,^38^ although less prevalent, are associated with high treatment costs and long-term disability.

We utilized patient-level hospital discharge data from California during 2005–2018 to examine lag effects and subgroup analyses based on age, sex, race, and ethnicity. Our findings aim to provide evidence of the short-term health effects of ambient air pollution on the nervous system, helping guide public health efforts and policies to reduce these risks.

## Methods

### Emergency department visits and air pollution data

We obtained patient-level hospital discharge data from the California Office of Statewide Health Planning and Development during the period 2005–2018. Our definition of ED visits included patients who were discharged from the ED, as well as those that led to hospital admission. ED records included the admission date, age at the visit, self-reported residential ZIP codes, race, ethnicity, and International Classification of Diseases version 9/10 (ICD-9/10) diagnosis codes. Using both primary and secondary diagnosis codes, we identified visits with at least one of the following four nervous system-related outcomes: PD (ICD-9 codes 332, ICD-10 codes G20, G21), MS (ICD-9 codes 340, ICD-10 codes G35), migraine (ICD-9 codes 346, ICD-10 codes G43), or seizures (ICD-9 codes 345, 780.39, ICD-10 codes G40, G41). The occurrence of PD in individuals under 18 is extremely rare, so we excluded PD-related ED visits for individuals under the age of 18.

ED visit data were linked to daily estimated air pollution concentrations at the ZIP-code level using the date of visit and patients’ residential ZIP code. The daily air pollution data were based on bias-corrected outputs of the Community Multiscale Air Quality model, which provides pollutant concentrations at a 12-km spatial resolution.^39^ Bias correction was performed using observations from the air quality monitoring network and land use variables. These data were aggregated to the ZIP-code level by averaging grid cell values overlapping with each ZIP code. Daily specific humidity and average temperature were processed similarly using the 1-km High-resolution Urban Meteorology for Impacts Dataset.^40^

### Statistical analyses

Short-term associations between air pollutant concentrations and ED visits were estimated via a case-crossover design. For each ED visit, we defined visit-specific controls as the same day of the week falling in the same month, year, and ZIP codes. We examined lag effects by including air pollutant concentrations from the same day (lag 0) and the three previous days (lag 1, lag 2, and lag 3) in the model and focused on cumulative lag effects, which aligns with our research objective to capture the total impact of exposure over time and helps to reduce potential issues of collinearity among adjacent lag days. Due to the large sample size, we utilized conditional Poisson for computation with strata defined by ZIP code, day of week, month, and year. The conditional Poisson approach also has the advantage of allowing for overdispersion via the quasi-Poisson specification.^41^ Associations between each pollutant (PM_2.5_, CO, and NO_2_) and each ED visit outcome were analyzed separately.

Our models accounted for several time-varying confounders. To control within-month residual temporal trends, we used natural cubic splines on the day of the year (1, 2, …, 365 or 366 for leap years) with 6 degrees of freedom and indicators for federal holidays. Nonlinear associations for daily average temperature and specific humidity were specified as natural cubic splines with 6 degrees of freedom. We conducted several sensitivity analyses to evaluate the robustness of the results from our primary analysis. First, we assessed the impact of varying degrees of freedom (8, 10, and 12) for the day-of-year spline used to model the overall temporal trend. Second, we assessed associations of air pollutant concentrations separately at different cumulative lags (lag 0–1, lag 0–2, and lag 0–3) to investigate cumulative effects. To address potential residual confounding by temperature, we conducted additional analyses using a distributed lag nonlinear model approach, in which temperature was modeled using a cross-basis function including lags up to 3 days (lag 0–3). Risk ratios (RRs) are reported per interquartile range (IQR) increase in exposure. All data analyses were performed in R 4.3.1.^42^

We conducted stratified analyses based on age, sex, and race/ethnicity, respectively. Age groups were categorized as <18, 19–44, 45–64, and ≥65 years. Sex was stratified into male and female groups. Race and ethnicity were classified into six categories: Hispanic, non-Hispanic Black, non-Hispanic White, non-Hispanic Asian, non-Hispanic American Indian or Alaska Native, and other. Given that younger onset PD cases may also experience exacerbations of PD and/or comorbid conditions, we have elected to include them in the analysis. Finally, we examined associations separately for visits that were discharged from the ED and those that led to hospital admission.

## Results

Table 1 gives descriptive statistics of ED visits for PD, MS, migraine, and seizure stratified by age, sex, and race/ethnicity. Overall, this study included over 6 million ED visits (609,213 for PD, 250,027 for MS, 1,950,387 for migraine, and 3,281,504 for seizure). For ED visits of PD, more than 86% of the patients were aged 65 or older, while for MS, migraines, and seizures, the majority of patients were adults aged 18–64 years. For ED visits related to MS and migraines, female patients significantly outnumbered male patients. Table S1; https://links.lww.com/EE/A391 summarizes the distribution of ED visits identified for each outcome by primary and secondary discharge diagnosis codes and by hospitalization status. Most ED visits for PD (96.3%) and MS (85.8%) were ascertained based on secondary diagnoses. Across conditions, the proportion of ED visits that resulted in hospitalization varied substantially, with approximately 50% for PD, 41% for MS, 38% for seizure, and only 19% for migraine.

**Table 1.** Study population characteristics of ED visits for four neurological conditions in California (2005–2018)

	Parkinson	Multiple sclerosis	Seizures	Migraines
Overall	609,213	250,027	3,281,504	1,950,387
<18	122 (0.0%)	1,257 (0.5%)	380,160 (11.6%)	83,817 (4.3%)
18–44	5,800 (1.0%)	72,192 (28.9%)	1,171,058 (35.7%)	1,079,566 (55.4%)
45–64	75,841 (12.4%)	121,063 (48.4%)	1,051,428 (32.0%)	633,897 (32.5%)
>64	527,450 (86.6%)	55,515 (22.2%)	678,858 (20.7%)	153,107 (7.9%)
Female	249,459 (40.9%)	170,965 (68.4%)	1,505,183 (45.9%)	1,544,629 (79.2%)
Male	303,074 (49.7%)	61,872 (24.7%)	1,607,818 (49.0%)	338,234 (17.3%)
Missing	56,680 (9.3%)	17,190 (6.9%)	168,503 (5.1%)	338,234 (3.5%)
Hispanic	101,897 (16.7%)	34,600 (13.8%)	878,841 (26.8%)	514,113 (26.4%)
Non-Hispanic Black	27,936 (4.6%)	33,532 (13.4%)	510,220 (15.5%)	205,454 (10.5%)
Non-Hispanic White	397,502 (65.2%)	166,000 (66.4%)	1,576,760 (48.0%)	1,062,657 (54.5%)
Non-Hispanic Asian	53,458 (8.8%)	3,826 (1.5%)	131,056 (4.0%)	63,306 (3.2%)
Non-Hispanic American Indian or Alaska Native	1,498 (0.2%)	553 (0.2%)	12,743 (0.4%)	9,733 (0.5%)
Non-Hispanic other	16,048 (2.6%)	6,208 (2.5%)	87,010 (2.7%)	45,933 (2.4%)
Missing	10,874 (1.8%)	5,308 (2.1%)	84,874 (2.6%)	49,191 (2.5%)

Mean, standard deviation, and IQR for pollutant concentrations are presented in Table 2. The three pollutants are positively correlated, with the strongest correlation observed between CO and NO_2_ (Pearson correlation = 0.73) compared with 0.46 for PM_2.5_ and NO_2_ and 0.49 for PM_2.5_ and CO.

**Table 2.** Mean, standard deviation (SD), and IQR of daily ZIP-code level pollutant concentrations (n = 9,328,664 ZIP code days)

Pollutant	Mean	SD	IQR
1-hour carbon monoxide (CO) (ppm)	0.57	0.45	0.43
1-hour nitrogen dioxide (NO_2_) (ppb)	17.24	14.98	21.3
24-hour PM_2.5_ (µg/m^3^)	8.67	5.52	5.18

**Figure 1.**
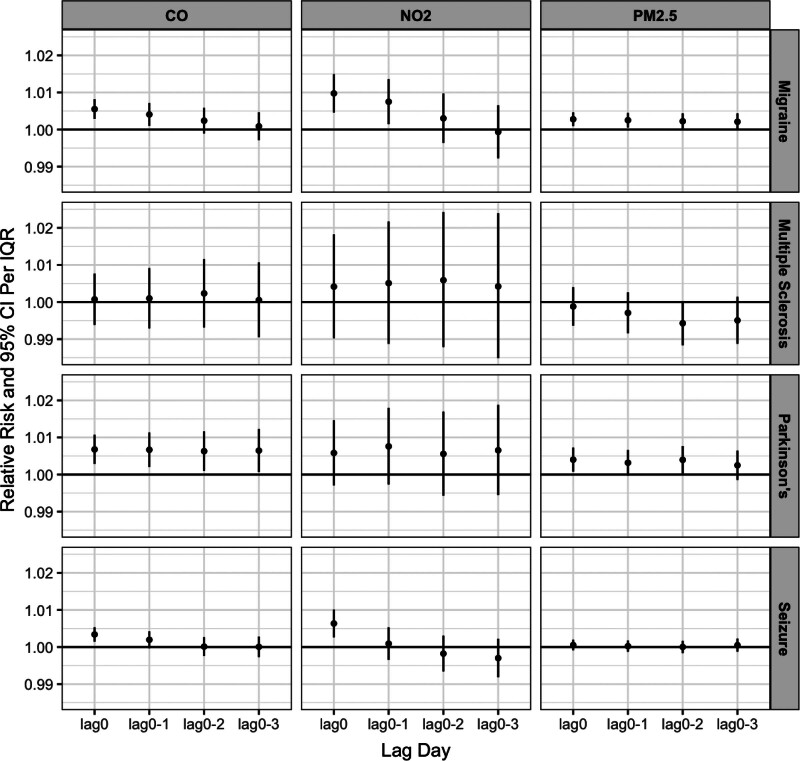
Short-term associations between ED visits for neurological conditions and per IQR changes in ambient air pollution levels in California, 2005–2018.

Figure 1 and Table S2; https://links.lww.com/EE/A391 present the estimated RR between ED visits and exposures to PM_2.5_, CO, and NO_2_ across different lag periods (lag 0 and cumulative lag up to 3 days). Overall, we observed positive associations between air pollutants and ED visits that varied by pollutants and outcomes. For PD, short-term association was strongest with CO exposure (lag 0 RR = 1.007, 95% confidence interval [CI]: 1.003, 1.011). We did not observe associations between MS and the three pollutants. For migraines, the strongest association per IQR increase in exposure was observed for NO_2_ exposure at lag 0 (lag 0 RR = 1.009, 95% CI: 1.005, 1.015), compared with CO (lag 0 RR = 1.006, 95% CI: 1.003, 1.008) and PM_2.5_ (lag 0 RR = 1.003, 95% CI: 1.001, 1.005). A decreasing trend in cumulative lag effects for migraines was observed with CO and NO_2_, whereas the lag effects with PM_2.5_ remained consistent. Similarly, associations for seizures were most prominent with NO_2_ exposure (lag 0 RR = 1.006, 95% CI: 1.003, 1.010) across different lag periods.

Sensitivity analysis was performed to evaluate the impact of degrees of freedom for temporal confounding (the day of the year). The results indicated that using 8, 10, and 12 degrees of freedom did not affect the short-term associations, supporting the robustness of our findings (Figure S1; https://links.lww.com/EE/A391). In addition, results from the temperature-lag sensitivity analysis (Figure S2; https://links.lww.com/EE/A391) were consistent with the primary analysis, showing no substantial changes in the estimated associations.

In analyses by ED visit disposition, estimates for PD, migraine, and seizures were broadly similar between patients who were admitted to the hospital and those who were discharged from the ED, with only slightly higher estimated NO_2_ effects among discharged PD ED visits. For MS, inpatient analyses suggested stronger positive associations with CO and NO_2_, whereas noninpatient results were closer to null (Figure S3; https://links.lww.com/EE/A391).

Table S4; https://links.lww.com/EE/A391 presents estimated RRs for same-day (lag 0) exposure from stratified analyses by sex, race/ethnicity, and age. For migraine ED visits, females had higher RRs for PM_2.5_ and CO. Older age groups exhibited stronger associations for migraines across all pollutants, particularly for CO and NO_2_ exposures. For MS, increased RRs were observed for NO_2_ and CO among non-Hispanic American Indian patients. Similarly, for seizures, associations with PM_2.5_ and CO were stronger in this population subgroup. Males exhibited higher RRs than females, especially for CO exposure. The pediatric MS group showed markedly higher RRs compared with other age groups. Hispanic patients showed weaker associations compared with non-Hispanic patients for CO and NO_2_. For PD ED visits, RRs for all three pollutants were higher among non-Hispanic American Indian patients compared with other racial/ethnic groups. Meanwhile, higher RRs were among male patients for all three pollutants. For seizures, patients under 18 showed stronger associations compared with other age groups, especially for CO and NO_2_ exposures.

## Discussion

We analyzed over 6 million ED visits related to four nervous system conditions, including PD, MS, migraines, and seizures, and evaluated their associations with short-term exposures to three air pollutants: PM_2.5_, CO, and NO_2_. This study represents the largest investigation of its kind in the United States and is unique due to the inclusion of both ED visits and acute hospitalization as the morbidity outcome. We found that same-day high ambient air pollution levels were associated with increased ED visits for migraine, PD, and seizures, but not for MS, except among those that led to hospitalization. This study adds to the growing epidemiological evidence that short-term exposure to ambient air pollution may affect the nervous system.

Furthermore, it is important to interpret our findings in the context of the clinical meaning of ED encounters. Previous literature has emphasized that ED visits for chronic neurological conditions such as PD, MS, and seizures typically represent acute exacerbations rather than incident cases.^43^ These exacerbations may be triggered or worsened by environmental exposures, aligning with the short-term nature of our study design.^44^ In addition, such visits are often influenced by coexisting chronic conditions, particularly cardiovascular, respiratory, and renal comorbidities,^45^ which themselves are sensitive to air pollution exposures.^46^ We examined other common chronic conditions, such as CVD, respiratory disease, or renal, that are co-diagnosed with the ED visit encounter. According to Table S3; https://links.lww.com/EE/A391, CVD, respiratory disease, and renal conditions were most common among PD ED visits with percentages of 74.77%, 31.39%, and 24.12%, respectively, and less frequent among patients with MS, seizures, and migraine.

Our study found associations between exposures to PM_2.5_, CO, and NO_2_ and the risk of PD-related ED visits. These results align with previous research indicating that air pollutants may exacerbate PD symptoms through pathways such as oxidative stress, neuroinflammation, and vascular dysfunction.^47^ Our subgroup analysis indicated that the magnitude of association was largest among non-Hispanic American Indians, likely reflecting a combination of socioeconomic disparities and a greater prevalence of comorbidities that may interact with PD.^48^ However, given the relatively small sample size in this group (n = 1,498; 0.2% of the total PD sample), these findings should be interpreted with caution. Furthermore, we found that males are more affected than females, which aligns with results from a previous study in Seoul,^49^ potentially due to biological differences, such as heightened dopaminergic neuron susceptibility, as well as occupational exposures and lifestyle factors that increase contact with air pollutants.^50^

Migraine is the most frequent neurological condition encountered in primary care.^51^ We found that short-term increases in PM_2.5_, CO, and NO_2_ levels were associated with elevated risks of migraine-related ED visits, particularly within 0–2 days after the exposure. Furthermore, elderly individuals were identified as being more susceptible to the effects of these pollutants. Although a definitive biomedical mechanism linking migraine to air pollution has not yet been established, emerging evidence suggests that inflammation and oxidative stress play crucial roles in the adverse neurological effects of air pollution.^52^ Our findings agree with the results of previous studies on the short-term effects of air pollution on migraine disease, especially among vulnerable age populations.^22,53^

The associations observed between lag 0 exposure to CO and NO_2_ and the risk of seizure-related ED visits suggest that the acute effects of air pollution may pose a health risk to individuals with epilepsy. Notably, non-Hispanic American Indians and young people (under 18) are more vulnerable compared with other racial and age groups.^48^ For children and adolescents, the developing brain is particularly susceptible to neuroinflammatory and neurotoxic damage caused by pollutants, potentially amplifying their risk of seizure episodes.^54^ The null associations with PM_2.5_ warrant further research and could explore longer lag periods or examine how chronic PM_2.5_ exposure interacts with seizure susceptibility, particularly in high-pollution regions.^55^

We did not observe overall associations between air pollutant exposure and MS ED visits, which is consistent with prior findings in Spain that there was no association between pollutants (PM_2.5_, CO, and NO_2_) and MS admissions.^56^ However, in subgroup analysis, we found that individuals under the age of 18 years with MS were more affected by the three pollutants, highlighting the vulnerability of younger populations with MS. But these findings should be interpreted with caution, given the small number of visits in the pediatric MS subgroup, which may reduce the stability of the estimates. Further investigation is needed to evaluate the robustness of these findings.

For PD and MS, ED visits are more likely to reflect exacerbations of comorbid conditions rather than incident cases. Prior literature has shown that PD patients frequently present to the ED due to falls, infections, cardiovascular complications, or neuropsychiatric symptoms.^43^ Similarly, MS patients have elevated risks of ED encounters and hospitalizations related to infections and respiratory conditions.^57^ These findings are consistent with our observation that PD and MS were mostly identified through secondary diagnoses in our dataset.

Our study has several strengths. First, our exposure data leveraged both monitoring measurements and numerical model simulations. This provides improved spatial and temporal coverage than relying only on monitor data. Second, we utilized statewide hospital discharge data that included patients of all ages, all insurance statuses, and both urban and rural neighborhoods. This enhances the reliability of our risk estimates and increases the applicability of the results to broader populations. Third, we evaluated neurological morbidity using ED visits, which is an important and understudied outcome. Finally, our study has a large sample size, which allowed us to separately examine associations across different demographic groups.

There are also some limitations in this study. First, ED visit outcomes were defined based on ICD codes from discharge records that are less accurate than medical records or adjudicated outcomes from prospective cohort studies. Second, we used temporal variations in outdoor air pollution levels as a proxy for the variations in personal exposure levels. While this could introduce measurement errors, we note that previous studies have shown that the effect of this kind of measurement error often leads to attenuated estimated relative risks.^58,59^ Case ascertainment is a further limitation of our study because of data limitations, as misclassification of neurological diseases in ED visits may occur.^60^ Although ED data provide larger numbers and greater power, diagnostic accuracy is generally lower than for inpatient records, as has been shown for cardiovascular outcomes.^61^

In summary, this study found that short-term exposure to PM_2.5_, CO, and NO_2_ was associated with increased ED visits for migraine, PD, and seizures, but not for MS. By examining several neurological conditions within one large population-based study in California, our results contribute to the growing evidence that air pollution may influence neurological health. Stratified analyses further suggest that the effects may vary across population subgroups, pointing to the importance of considering differential susceptibility in future research and public health strategies.

## Conflicts of interest statement

The authors declare that they have no conflicts of interest with regard to the content of this report.

## ACKNOWLEDGMENTS

We are grateful for the support of the health data source: California Department of Health Care Access and Information. Authorization to release this information does not imply endorsement of this study or its findings by any of this data source. The data source, their employees, officers, and agents make no representation, warranty, or guarantee as to the accuracy, completeness, currency, or suitability of the information provided here.

## Supplementary Material


