# Supplementary material for: Short-term associations between ambient air pollution and emergency department visits for Parkinson disease, multiple sclerosis, migraine, and seizure in California, 2005–2018
Source: Environ Epidemiol. 2025 Dec 23;10(1):e444. doi: 10.1097/EE9.0000000000000444 (PMC12737864; doi:10.1097/EE9.0000000000000444)
Supplement: Supplementary file 1 [file ee9-10-e444-s001.pdf]

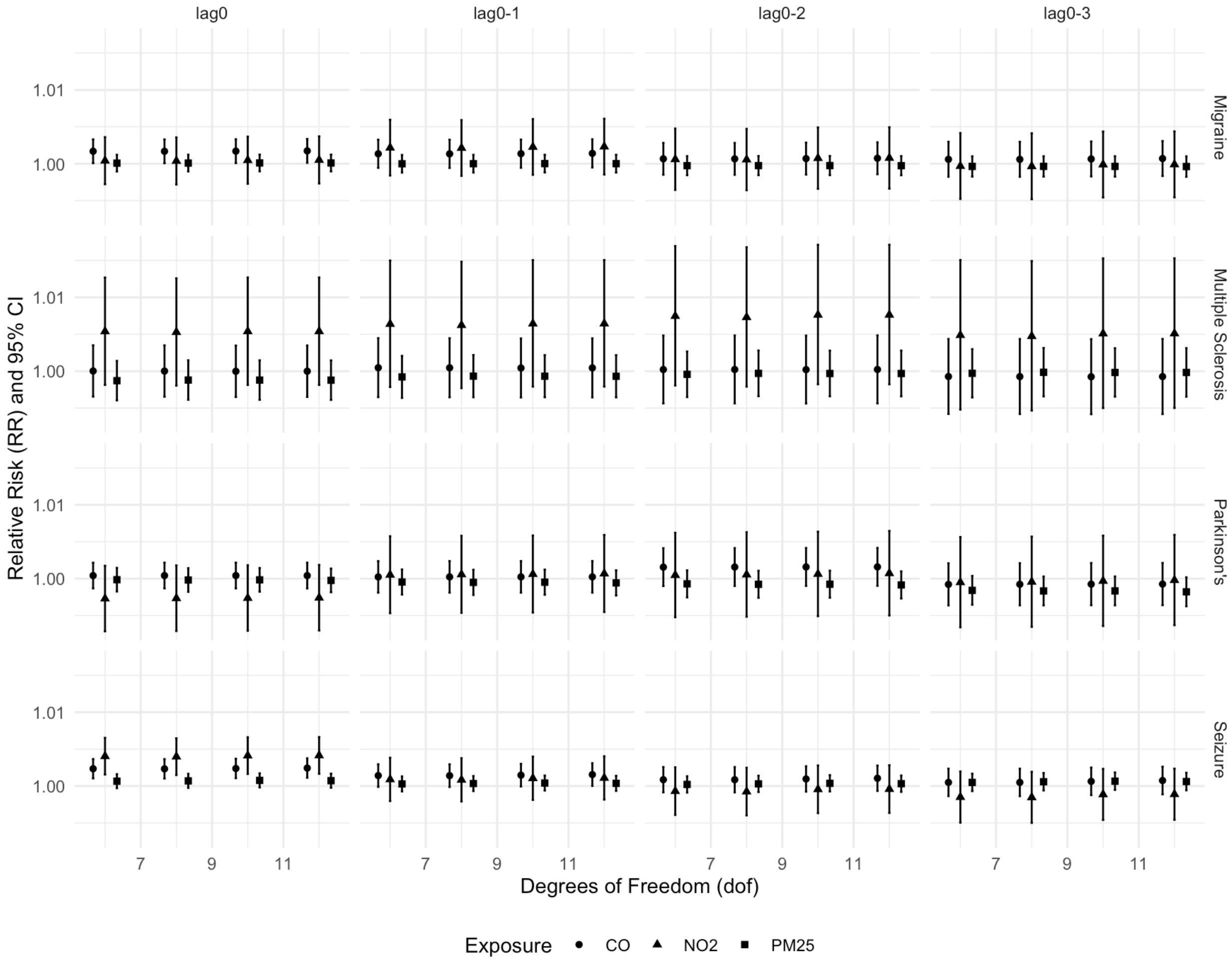

Figure S1. Sensitivity analysis of RR estimates obtained from models with different degrees of freedom for the day of year

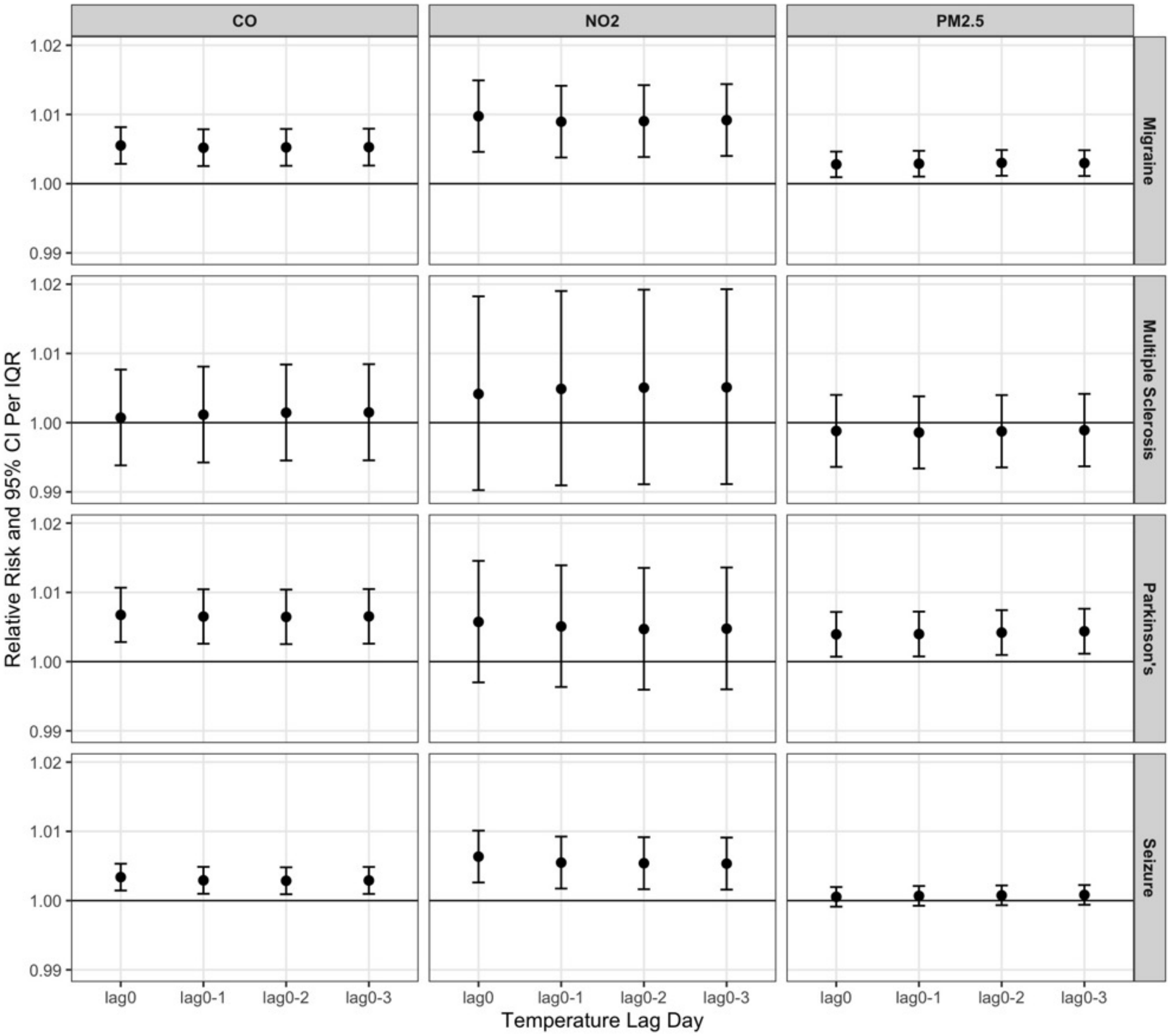

Figure S2. Sensitivity analysis of RR estimates for temperature lag effects (lag 0–3) using DLNM

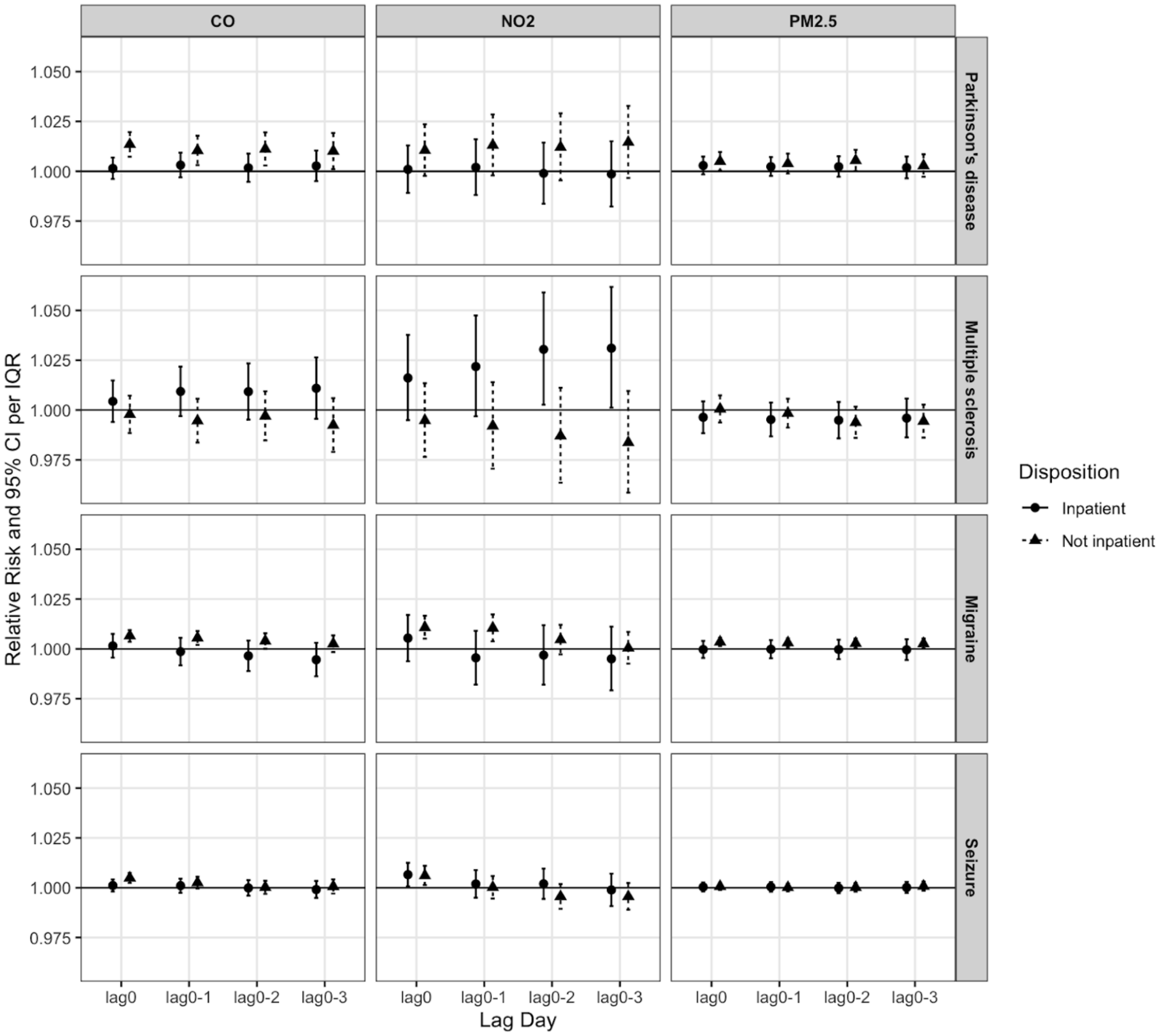

Figure S3. RR estimates of pollutants for ED visits by hospitalization status and lag day across neurological outcomes.

Table S1. Number of ED visits (percentage) for four neurological outcomes in California (2005-2018), classified by primary and secondary discharge diagnosis codes and by hospitalization status.

	Parkinson ‘s's	Migraines	Multiple Sclerosis	Seizures
Primary	25,112 (3.73%)	1,160,290 (54.81%)	38,466 (14.10%)	1,276,659 (36.65%)
Secondary	647,606 (96.27%)	956,823 (45.19%)	234,246 (85.90%)	2,206,437 (63.35%)
Hospitalization	337,556 (50.18%)	110,919 (40.67%)	393,967 (18.61%)	1,332,736 (38.26%)
Discharged	335,162 (49.82%)	161,793 (59.33%)	1,723,146 (81.39%)	2,150,360 (61.74%)
Total	672,718	2,117,113	272,712	3,483,096

Table S2. RR for nervous system disease-related ED visits associated with air pollution exposures across lag periods (lag0-lag3)

Outcome	Exposure	Lag	RR per IQR change	95%CI
Parkinson‘s	PM2.5	lag0	1.004	(1.001, 1.007)
Parkinson‘s	PM2.5	lag0-1	1.003	(1.000, 1.007)
Parkinson‘s	PM2.5	lag0-2	1.004	(1.000, 1.008)
Parkinson‘s	PM2.5	lag0-3	1.002	(0.999, 1.006)
Parkinson‘s	CO	lag0	1.007	(1.003, 1.011)
Parkinson‘s	CO	lag0-1	1.007	(1.002, 1.011)
Parkinson‘s	CO	lag0-2	1.006	(1.001, 1.012)
Parkinson‘s	CO	lag0-3	1.006	(1.001, 1.012)
Parkinson‘s	NO2	lag0	1.006	(0.997, 1.015)
Parkinson‘s	NO2	lag0-1	1.008	(0.997, 1.018)
Parkinson‘s	NO2	lag0-2	1.006	(0.994, 1.017)
Parkinson‘s	NO2	lag0-3	1.007	(0.994, 1.019)
Multiple Sclerosis	PM2.5	lag0	0.999	(0.994, 1.004)
Multiple Sclerosis	PM2.5	lag0-1	0.997	(0.992, 1.003)
Multiple Sclerosis	PM2.5	lag0-2	0.994	(0.988, 1.000)
Multiple Sclerosis	PM2.5	lag0-3	0.995	(0.989, 1.001)
Multiple Sclerosis	CO	lag0	1.001	(0.994, 1.008)
Multiple Sclerosis	CO	lag0-1	1.001	(0.993, 1.009)
Multiple Sclerosis	CO	lag0-2	1.002	(0.993, 1.012)
Multiple Sclerosis	CO	lag0-3	1.001	(0.991, 1.011)
Multiple Sclerosis	NO2	lag0	1.004	(0.990, 1.018)
Multiple Sclerosis	NO2	lag0-1	1.005	(0.989, 1.022)
Multiple Sclerosis	NO2	lag0-2	1.006	(0.988, 1.024)
Multiple Sclerosis	NO2	lag0-3	1.004	(0.985, 1.024)
Migraines	PM2.5	lag0	1.003	(1.001, 1.005)
Migraines	PM2.5	lag0-1	1.003	(1.001, 1.004)
Migraines	PM2.5	lag0-2	1.002	(1.000, 1.004)
Migraines	PM2.5	lag0-3	1.002	(1.000, 1.004)
Migraines	CO	lag0	1.006	(1.003, 1.008)
Migraines	CO	lag0-1	1.004	(1.001, 1.007)
Migraines	CO	lag0-2	1.002	(0.999, 1.006)
Migraines	CO	lag0-3	1.001	(0.997, 1.005)
Migraines	NO2	lag0	1.010	(1.005, 1.015)
Migraines	NO2	lag0-1	1.008	(1.001, 1.014)
Migraines	NO2	lag0-2	1.003	(0.996, 1.010)
Migraines	NO2	lag0-3	0.999	(0.992, 1.007)
Seizures	PM2.5	lag0	1.001	(0.999, 1.002)
Seizures	PM2.5	lag0-1	1.000	(0.999, 1.002)
Seizures	PM2.5	lag0-2	1.000	(0.998, 1.002)
Seizures	PM2.5	lag0-3	1.001	(0.999, 1.002)
Seizures	CO	lag0	1.003	(1.001, 1.005)
Seizures	CO	lag0-1	1.002	(1.000, 1.004)
Seizures	CO	lag0-2	1.000	(0.998, 1.003)

Seizures	CO	lag0-3	1.000 (0.997, 1.003)
Seizures	NO2	lag0	1.006 (1.003, 1.010)
Seizures	NO2	lag0-1	1.001 (0.997, 1.005)
Seizures	NO2	lag0-2	0.998 (0.993, 1.003)
Seizures	NO2	lag0-3	0.997 (0.992, 1.002)

Table S3. The proportion of cause-specific ED visits with a co-diagnosis for Cardiovascular disease (CVD), Respiratory disease (RESP) or Renal

**Condition**	**CVD (%)**	**RESP (%)**	**Renal (%)**
**Parkinson's**	74.77%	31.39%	24.12%
**Multiple Sclerosis**	42.36%	23.07%	11.66%
**Seizures**	40.54%	25.33%	12.56%
**Migraines**	26.96%	18.64%	5.63%

Table S4. Stratified analysis of ED visits for nervous system-related diseases and air pollution exposure by demographic and age groups

Outcome	Exposure	Stratification	ED Visit Counts	RR per IQR change	95%CI	RR Ratio	p-value
Migraines	PM2.5	Overall	2,117,113	1.003	(1.001, 1.005)	--	--
Migraines	PM2.5	Female	1,679,318	1.004	(1.002, 1.006)	REF	REF
Migraines	PM2.5	Male	370,294	1	(0.998, 1.003)	0.996	0.028
Migraines	PM2.5	Hispanic	568,931	1.003	(1.001, 1.005)	1.001	0.726
Migraines	PM2.5	Non-Hispanic Black	223,176	1.008	(1.005, 1.010)	1.005	0.001
Migraines	PM2.5	Non-Hispanic White	1,139,593	1.003	(1.000, 1.005)	REF	REF
Migraines	PM2.5	Non-Hispanic Asian	71,842	1.002	(1.000, 1.005)	1.000	0.938
Migraines	PM2.5	Non-Hispanic American Indian	10,503	1.006	(1.003, 1.009)	1.004	0.041
Migraines	PM2.5	Non-Hispanic Other	51,357	0.98	(0.977, 0.983)	0.977	0.000
Migraines	PM2.5	<18	91,054	1.008	(1.005, 1.011)	1.006	<0.001
Migraines	PM2.5	19-44	1,161,781	1.002	(1.000, 1.004)	REF	REF
Migraines	PM2.5	45-64	687,891	1.003	(1.000, 1.005)	1.001	0.556
Migraines	PM2.5	>64	176,387	1.007	(1.004, 1.009)	1.005	0.002
Migraines	NO2	Overall	2,117,113	1.01	(1.005, 1.015)	--	--
Migraines	NO2	Female	1,679,318	1.009	(1.003, 1.014)	REF	REF
Migraines	NO2	Male	370,294	1.013	(1.006, 1.020)	1.004	0.330
Migraines	NO2	Hispanic	568,931	1.01	(1.004, 1.016)	1.001	0.745
Migraines	NO2	Non-Hispanic Black	223,176	1.018	(1.012, 1.024)	1.009	0.030
Migraines	NO2	Non-Hispanic White	1,139,593	1.008	(1.002, 1.015)	REF	REF

Migraines	NO2	Non-Hispanic Asian	71,842	1.009	(1.002, 1.016)	1.000	0.961
Migraines	NO2	Non-Hispanic American Indian	10,503	1.026	(1.017, 1.035)	1.017	0.002
Migraines	NO2	Non-Hispanic Other	51,357	0.988	(0.981, 0.996)	0.980	<0.001
Migraines	NO2	<18	91,054	1.004	(0.997, 1.012)	0.993	0.135
Migraines	NO2	19-44	1,161,781	1.012	(1.006, 1.017)	REF	REF
Migraines	NO2	45-64	687,891	1.005	(0.999, 1.012)	0.994	0.155
Migraines	NO2	>64	176,387	1.017	(1.010, 1.025)	1.005	0.256
Migraines	CO	Overall	2,117,113	1.006	(1.003, 1.008)	--	--
Migraines	CO	Female	1,679,318	1.006	(1.003, 1.009)	REF	REF
Migraines	CO	Male	370,294	1.004	(1.001, 1.008)	0.998	0.426
Migraines	CO	Hispanic	568,931	1.005	(1.002, 1.008)	1.000	0.920
Migraines	CO	Non-Hispanic Black	223,176	1.011	(1.008, 1.014)	1.006	0.005
Migraines	CO	Non-Hispanic White	1,139,593	1.005	(1.002, 1.008)	REF	REF
Migraines	CO	Non-Hispanic Asian	71,842	0.996	(0.993, 1.000)	0.991	<0.001
Migraines	CO	Non-Hispanic American Indian	10,503	0.991	(0.987, 0.996)	0.986	<0.001
Migraines	CO	Non-Hispanic Other	51,357	0.992	(0.988, 0.996)	0.988	<0.001
Migraines	CO	<18	91,054	1.006	(1.002, 1.010)	1.000	0.986
Migraines	CO	19-44	1,161,781	1.006	(1.003, 1.009)	REF	REF

Migraines	CO	45-64	687,891	1.004	(1.000, 1.007)	0.998	0.351
Migraines	CO	>64	176,387	1.011	(1.007, 1.015)	1.005	0.029
Multiple Sclerosis	PM2.5	Overall	272,712	0.999	(0.994, 1.004)	--	--
Multiple Sclerosis	PM2.5	Female	187,420	0.997	(0.991, 1.002)	REF	REF
Multiple Sclerosis	PM2.5	Male	68,103	1.003	(0.998, 1.009)	1.006	0.105
Multiple Sclerosis	PM2.5	Hispanic	38,543	0.999	(0.993, 1.004)	1.000	0.934
Multiple Sclerosis	PM2.5	Non-Hispanic Black	36,722	0.998	(0.993, 1.003)	0.999	0.769
Multiple Sclerosis	PM2.5	Non-Hispanic White	179,731	0.999	(0.994, 1.004)	REF	REF
Multiple Sclerosis	PM2.5	Non-Hispanic Asian	4,261	0.989	(0.984, 0.993)	0.990	0.005
Multiple Sclerosis	PM2.5	Non-Hispanic American Indian	653	0.937	(0.931, 0.943)	0.938	<0.001
Multiple Sclerosis	PM2.5	Non-Hispanic Other	7,141	0.986	(0.980, 0.992)	0.987	0.001
Multiple Sclerosis	PM2.5	<18	1,365	1.027	(1.021, 1.033)	1.030	<0.001
Multiple Sclerosis	PM2.5	19-44	78,256	1.006	(1.001, 1.012)	1.009	0.020
Multiple Sclerosis	PM2.5	45-64	130,734	0.997	(0.992, 1.002)	REF	REF
Multiple Sclerosis	PM2.5	>64	62,357	0.993	(0.988, 0.998)	0.996	0.296
Multiple Sclerosis	NO2	Overall	272,712	1.004	(0.990, 1.018)	--	--
Multiple Sclerosis	NO2	Female	187,420	1.004	(0.990, 1.018)	REF	REF
Multiple Sclerosis	NO2	Male	68,103	1.007	(0.992, 1.022)	1.003	0.773
Multiple Sclerosis	NO2	Hispanic	38,543	0.991	(0.977, 1.005)	0.982	0.081

Multiple Sclerosis	NO2	Non-Hispanic Black	36,722	0.995	(0.983, 1.008)	0.986	0.168
Multiple Sclerosis	NO2	Non-Hispanic White	179,731	1.009	(0.994, 1.024)	REF	REF
Multiple Sclerosis	NO2	Non-Hispanic Asian	4,261	1.036	(1.021, 1.050)	1.026	0.012
Multiple Sclerosis	NO2	Non-Hispanic American Indian	653	1.25	(1.229, 1.271)	1.238	<0.001
Multiple Sclerosis	NO2	Non-Hispanic Other	7,141	0.992	(0.977, 1.007)	0.983	0.106
Multiple Sclerosis	NO2	<18	1,365	1.078	(1.063, 1.094)	1.082	<0.001
Multiple Sclerosis	NO2	19-44	78,256	1.015	(1.000, 1.029)	1.018	0.085
Multiple Sclerosis	NO2	45-64	130,734	0.997	(0.982, 1.011)	REF	REF
Multiple Sclerosis	NO2	>64	62,357	1.006	(0.991, 1.021)	1.009	0.375
Multiple Sclerosis	CO	Overall	272,712	1.001	(0.994, 1.008)	--	--
Multiple Sclerosis	CO	Female	187,420	0.998	(0.991, 1.006)	REF	REF
Multiple Sclerosis	CO	Male	68,103	1.007	(1.000, 1.014)	1.009	0.095
Multiple Sclerosis	CO	Hispanic	38,543	0.995	(0.987, 1.003)	0.995	0.388
Multiple Sclerosis	CO	Non-Hispanic Black	36,722	1.003	(0.996, 1.010)	1.003	0.507
Multiple Sclerosis	CO	Non-Hispanic White	179,731	0.999	(0.992, 1.007)	REF	REF
Multiple Sclerosis	CO	Non-Hispanic Asian	4,261	1.009	(1.001, 1.016)	1.009	0.089
Multiple Sclerosis	CO	Non-Hispanic American Indian	653	1.136	(1.125, 1.147)	1.136	0.000
Multiple Sclerosis	CO	Non-Hispanic Other	7,141	1.01	(1.004, 1.015)	1.010	0.026

Multiple Sclerosis	CO	<18	1,365	1.018	(1.010, 1.027)	1.021	<0.001
Multiple Sclerosis	CO	19-44	78,256	1.006	(0.999, 1.014)	1.010	0.072
Multiple Sclerosis	CO	45-64	130,734	0.997	(0.990, 1.004)	REF	REF
Multiple Sclerosis	CO	>64	62,357	1.003	(0.996, 1.011)	1.006	0.214
Parkinson‘s	PM2.5	Overall	672,718	1.004	(1.001, 1.007)	--	--
Parkinson‘s	PM2.5	Female	276,108	1.003	(0.999, 1.006)	0.999	0.740
Parkinson‘s	PM2.5	Male	340,016	1.003	(1.000, 1.007)	REF	REF
Parkinson‘s	PM2.5	Hispanic	113,427	1.006	(1.002, 1.009)	1.002	0.358
Parkinson‘s	PM2.5	Non-Hispanic Black	30,791	0.996	(0.993, 1.000)	0.993	0.005
Parkinson‘s	PM2.5	Non-Hispanic White	436,481	1.003	(1.000, 1.007)	REF	REF
Parkinson‘s	PM2.5	Non-Hispanic Asian	60,124	1.006	(1.003, 1.009)	1.003	0.262
Parkinson‘s	PM2.5	Non-Hispanic American Indian	1,684	1.05	(1.046, 1.053)	1.046	<0.00
Parkinson‘s	PM2.5	Non-Hispanic Other	18,405	1	(0.996, 1.003)	0.996	0.172
Parkinson‘s	PM2.5	19-44	6,343	0.976	(0.973, 0.980)	0.973	<0.001
Parkinson‘s	PM2.5	45-64	83,017	1.008	(1.004, 1.012)	1.004	0.084
Parkinson‘s	PM2.5	>64	583,358	1.004	(1.000, 1.007)	REF	REF
Parkinson‘s	NO2	Overall	672,718	1.006	(0.997, 1.015)	--	--
Parkinson‘s	NO2	Female	276,108	0.996	(0.987, 1.006)	0.983	0.012
Parkinson‘s	NO2	Male	340,016	1.013	(1.004, 1.023)	REF	REF

Parkinson's	NO2	Hispanic	113,427	1.013	(1.004, 1.023)	1.012	0.068
Parkinson's	NO2	Non-Hispanic Black	30,791	0.998	(0.990, 1.007)	0.997	0.663
Parkinson's	NO2	Non-Hispanic White	436,481	1.001	(0.992, 1.011)	REF	REF
Parkinson's	NO2	Non-Hispanic Asian	60,124	1.023	(1.013, 1.032)	1.022	0.001
Parkinson's	NO2	Non-Hispanic American Indian	1,684	1.218	(1.205, 1.231)	1.217	<0.001
Parkinson's	NO2	Non-Hispanic Other	18,405	0.986	(0.976, 0.996)	0.985	0.027
Parkinson's	NO2	19-44	6,343	1.044	(1.033, 1.054)	1.039	<0.001
Parkinson's	NO2	45-64	83,017	1.013	(1.003, 1.023)	1.008	0.212
Parkinson's	NO2	>64	583,358	1.004	(0.995, 1.013)	REF	REF
Parkinson's	CO	Overall	672,718	1.007	(1.003, 1.011)	--	--
Parkinson's	CO	Female	276,108	1.006	(1.001, 1.010)	0.996	0.262
Parkinson's	CO	Male	340,016	1.01	(1.005, 1.014)	REF	REF
Parkinson's	CO	Hispanic	113,427	1.014	(1.008, 1.019)	1.009	0.005
Parkinson's	CO	Non-Hispanic Black	30,791	1.005	(1.000, 1.009)	1.000	0.953
Parkinson's	CO	Non-Hispanic White	436,481	1.004	(1.001, 1.008)	REF	REF
Parkinson's	CO	Non-Hispanic Asian	60,124	1.016	(1.011, 1.021)	1.011	<0.001
Parkinson's	CO	Non-Hispanic American Indian	1,684	1.123	(1.116, 1.130)	1.118	<0.001
Parkinson's	CO	Non-Hispanic Other	18,405	1.008	(1.003, 1.013)	1.004	0.255

Parkinson's	CO	19-44	6,343	0.98	(0.975, 0.985)	0.975	<0.001
Parkinson's	CO	45-64	83,017	1.018	(1.013, 1.024)	1.013	<0.001
Parkinson's	CO	>64	583,358	1.005	(1.001, 1.009)	REF	REF
Seizures	PM2.5	Overall	3,483,096	1.001	(0.999, 1.002)	--	--
Seizures	PM2.5	Female	1,602,811	1	(0.998, 1.002)	0.999	0.563
Seizures	PM2.5	Male	1,711,850	1.001	(0.999, 1.003)	REF	REF
Seizures	PM2.5	Hispanic	941,384	1.001	(1.000, 1.003)	1.002	0.224
Seizures	PM2.5	Non-Hispanic Black	542,013	1	(0.998, 1.001)	1.000	0.913
Seizures	PM2.5	Non-Hispanic White	1,662,286	1	(0.998, 1.002)	REF	REF
Seizures	PM2.5	Non-Hispanic Asian	140,711	1.002	(1.000, 1.005)	1.003	0.059
Seizures	PM2.5	Non-Hispanic American Indian	13,711	1.02	(1.018, 1.023)	1.021	<0.001
Seizures	PM2.5	Non-Hispanic Other	95,001	1	(0.997, 1.002)	1.000	0.895
Seizures	PM2.5	<18	400,390	1.002	(1.000, 1.004)	1.003	0.077
Seizures	PM2.5	19-44	1,239,895	0.999	(0.998, 1.001)	REF	REF
Seizures	PM2.5	45-64	1,112,563	1.003	(1.001, 1.005)	1.003	0.012
Seizures	PM2.5	>64	730,248	0.998	(0.996, 1.000)	0.999	0.409
Seizures	NO2	Overall	3,483,096	1.006	(1.003, 1.010)	--	--
Seizures	NO2	Female	1,602,811	1.006	(1.001, 1.011)	0.999	0.880
Seizures	NO2	Male	1,711,850	1.007	(1.002, 1.011)	REF	REF

Seizures	NO2	Hispanic	941,384	1.003	(0.998, 1.008)	0.999	0.671
Seizures	NO2	Non-Hispanic Black	542,013	1.01	(1.005, 1.014)	1.005	0.113
Seizures	NO2	Non-Hispanic White	1,662,286	1.004	(1.000, 1.009)	REF	REF
Seizures	NO2	Non-Hispanic Asian	140,711	1.029	(1.023, 1.035)	1.025	<0.001
Seizures	NO2	Non-Hispanic American Indian	13,711	0.999	(0.992, 1.007)	0.995	0.256
Seizures	NO2	Non-Hispanic Other	95,001	1.016	(1.010, 1.023)	1.012	0.004
Seizures	NO2	<18	400,390	1.011	(1.005, 1.017)	1.010	0.013
Seizures	NO2	19-44	1,239,895	1.001	(0.996, 1.006)	REF	REF
Seizures	NO2	45-64	1,112,563	1.008	(1.003, 1.013)	1.006	0.073
Seizures	NO2	>64	730,248	1.011	(1.005, 1.016)	1.009	0.013
Seizures	CO	Overall	3,483,096	1.003	(1.001, 1.005)	--	--
Seizures	CO	Female	1,602,811	1.004	(1.002, 1.006)	1.001	0.478
Seizures	CO	Male	1,711,850	1.003	(1.000, 1.005)	REF	REF
Seizures	CO	Hispanic	941,384	1.002	(0.999, 1.004)	0.998	0.348
Seizures	CO	Non-Hispanic Black	542,013	1.003	(1.001, 1.006)	1.000	0.922
Seizures	CO	Non-Hispanic White	1,662,286	1.003	(1.001, 1.006)	REF	REF
Seizures	CO	Non-Hispanic Asian	140,711	1.011	(1.008, 1.014)	1.008	<0.001
Seizures	CO	Non-Hispanic American Indian	13,711	1.024	(1.021, 1.028)	1.021	<0.001

Seizures	CO	Non-Hispanic Other	95,001	1.002	(0.999, 1.006)	0.999	0.540
Seizures	CO	<18	400,390	1.007	(1.004, 1.010)	1.007	<0.001
Seizures	CO	19-44	1,239,895	1	(0.997, 1.003)	REF	REF
Seizures	CO	45-64	1,112,563	1.005	(1.003, 1.008)	1.005	0.003
Seizures	CO	>64	730,248	1.004	(1.001, 1.007)	1.004	0.041
